# Clinical and Radiological Features of Type A Aortic Dissection: A Retrospective Observational Study—An Indian Perspective

**DOI:** 10.1055/a-2839-9907

**Published:** 2026-04-06

**Authors:** Amitabh Satsangi, Surabhi Puri, Sumanth Raghuprakash, V. Devagourou

**Affiliations:** 1 Department of Cardio Thoracic and Vascular Surgery (CTVS), All India Institute of Medical Sciences, New Delhi, India; 2 Department of Community Medicine, Maulana Azad Medical College, New Delhi, India; 3 Department of Cardio Thoracic and Vascular Surgery (CTVS), Kasturba Medical College Mangalore, Karnataka, India

**Keywords:** acute cardiac care, cardiac computed tomography angiography, radiological features, demographics, Type A aortic dissection

## Abstract

**Introduction:**

Acute aortic dissection remains a hazardous and unpredictable condition. Although it is the most common acute aortic disease requiring surgical intervention, still up to 35% of patients are misdiagnosed on the initial presentation. Presentation of aortic dissection is protean, contributing to confusion in diagnosis and a delay in appropriate, timely intervention. Early and accurate diagnosis and treatment are crucial for survival. The clinical diagnosis of aortic dissection depends on awareness of the entity, keen clinical suspicion, and an understanding of its varied manifestations. Unfortunately, many patients die before they can receive hospital treatment.

**Materials and Methods:**

A total number of 208 patients diagnosed with Type A aortic dissection (TAAD) and operated on in our center from June 2014 to June 2019 were included in the study. After review of the medical records of 178 patients, data were available for 162 patients. The aim was to delineate presentation patterns, various clinical manifestations, and radiological features of Stanford TAAD. Univariate analysis was used to provide frequency distribution and percentage distribution of qualitative demographic and comorbidity variables. Mean ± standard deviation was reported, whereas for non-normally distributed quantitative variables, median ± (min–max) was reported. Data analysis was performed using the Statistical Package for Social Sciences, version 18.0 (SPSS, Chicago, IL).

**Results:**

A total of 162 patient records were reviewed; 131 (80.8%) patients were male, and 31 (19.2%) were female. The mean age of all patients was 43.3 years (± 13.49). A total of 146 patients (90.1%) had TAAD, and 16 patients (9.8%) had chronic TAAD. Severe pain was the most common presenting symptom, and 88.9% of patients reported chest pain. Four patients (2.7%) had renal malperfusion, and 23 (14%) had lower limb ischemia, with three having bilateral lower limb ischemia. Five patients had cerebrovascular accidents of new onset on presentation. Chest radiography showed mediastinal widening in 143 patients (88.8%). Two-dimensional (2D) echocardiography patients (87%) had dilated ascending aortas, with dissection flaps seen in 131 (81.4%) patients. Computed tomography (CT) dimensions–aortic valve annulus mean diameter was 31.5 mm (± 7.1), with the maximum diameter being 50 mm and minimum diameter being 16 mm.

**Conclusion:**

The clinical characteristics and radiological features of TAAD in the past 10 years in our center were analyzed. We also compared our data with those reported by International Registry for Aortic Dissection, Japan Registry of Aortic Dissection, and German Registry for Acute Aortic Dissection Type A. Acute chest pain was the most common presenting complaint. CT angiography was the investigation of choice, with TTE being a helpful supportive tool. The majority of our patients had TAAD despite aortic diameter being less than 55 mm. Our demographic data and clinical presentation were similar to Chinese data, owing to similar socio-economic backgrounds.

## Introduction

Aortic dissection occurs when there is a tear in the inner layer, allowing blood to enter through the tear and fill up between the inner and middle layers, causing these layers to separate or “dissect.” In 1760, King George II succumbed to catastrophic aortic dissection. Dr. Nicholls gave a detailed account of the incident, and surprisingly, to this date, aortic dissection still has a similar enigma surrounding itself. 1 Experts quote the incidence of aortic dissection as 5 to 30 patients per million people annually. 2

Acute aortic dissection remains a hazardous and unpredictable condition. Despite being the most common acute aortic disease requiring surgical intervention, up to 35% of patients receive a false diagnosis upon initial presentation. 3 Presentation of aortic dissection is protean, contributing to confusion in diagnosis and a delay in appropriate, timely intervention. Early and accurate diagnosis and treatment are crucial for survival. The clinical diagnosis of aortic dissection depends on an awareness of the entity, keen clinical suspicion, and an understanding of the varied manifestations. Unfortunately, many patients die before they can receive hospital treatment. An estimated 21% of patients with aortic dissection pass away before receiving hospital treatment. 4

During the initial 48 hours, patients with acute Type A aortic dissection (TAAD) have a mortality rate of 1 to 2% per hour. 5 Acute aortic dissection poses a unique challenge for physicians because of its relative rarity and its ability to mimic other medical conditions. With advances in diagnostic methods such as computed tomography (CT) angiography and 2D echocardiography, diagnosing acute aortic dissection has become much easier and more accurate. However, it still requires an apt clinical suspicion. 6 Diagnosis is delayed more than 24 hours after an initial presentation in almost half of all cases, highlighting the need for emergency physicians to maintain reasonable clinical suspicion for acute aortic dissection in patients with chest, back, and abdominal pain. 7

With early diagnosis and optimal medical and surgical therapy, 30-day survival can exceed 80%. 8 The Indian scenario itself is plagued by problems of correct, timely diagnosis, and early referral of such extremely critical cases, whose prognosis entirely depends upon the time lapse between the diagnosis and intervention. There is a lack of awareness of the condition and the gravity of the situation. It necessitates looking into the clinical features and the radiological features of patients already diagnosed as TAAD to develop resources that aid in early clinical suspicion and radiological detection.

## Materials and Methods

The institutional ethics committee granted ethical approval. A retrospective observational study was performed. The study aimed to assess the imaging features of Type A aortic dissection (TAAD) and describe the various symptoms and manifestations of acute TAAD in a specialized hospital.

A total of 208 patients with a diagnosis of TAAD who underwent surgery at our facility between June 2014 and June 2019 were included in the study. Data were available for 162 patients after 178 patients' medical records were reviewed. The objective was to outline the radiological characteristics, different clinical manifestations, and presentation patterns of Stanford TAAD.

We recorded the patients' demographic profile, including their age and sex. We recorded the month in which the patient presented. Presenting clinical features like pain, dyspnea, palpitation, and syncope were documented. We further categorized pain as a presenting clinical feature based on its type, duration, site, and severity. We further investigated the type, duration, site, and severity of the pain. Features like urological manifestations, neurological manifestations, gastrointestinal manifestations, and limb ischemia were also documented. We documented any significant past history suggestive of diabetes mellitus (DM), hypertension (HTN), or a known aortic aneurysm. We noted any personal history of addiction, past or present, including smoking and alcoholism. The physical examination recorded features such as blood pressure, cardiac murmur, peripheral pulse deficit, and malperfusion syndromes.

A suspected case of TAAD was noted following investigations, which are part of the routine protocol for patients admitted to the emergency department.

1. Chest X-ray posteroanteroposterior view.
2. Transthoracic echocardiography (TTE).
3. Electrocardiogram (ECG)-gated CT aortogram with coronary evaluation.

**Fig. 1:**
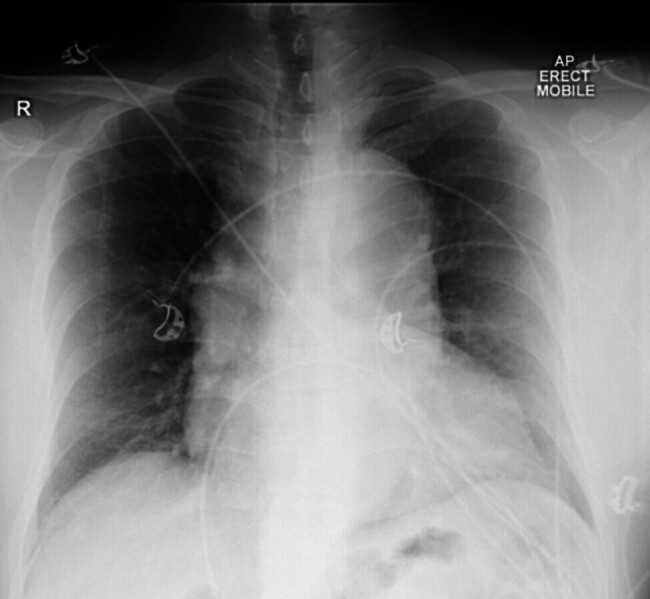
A chest X-Ray - Showing presence of mediastinal widening.

**Fig. 2:**
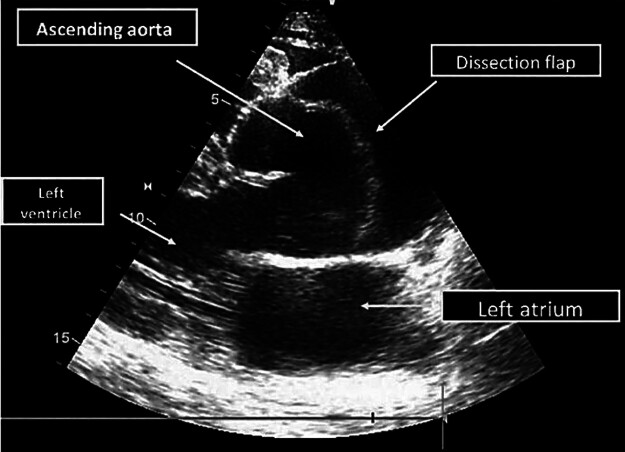
Two dimensional Echocardiography depicting dissection flap in ascending aorta.

In the chest X-ray, the presence or absence of mediastinal widening was recorded. ( Fig. 1 ) Maximal MW (mediastinal width) was defined as the longest distance from the right side to the left side of the upper mediastinum at the level of the aortic knob. A mediastinum measurement taken from a person lying down on an X-ray was considered widened, if it was 8 cm or more, or more than one-third of the distance across the chest at the level of the aortic knob. 9 10 In TTE, we measured the size of the ring at the aortic valve, checked if the beginning part of the ascending aorta was enlarged (if it was larger than 4.0 cm), 11 looked for any tears, examined the shape, and function of the valve, noted any fluid around the heart, and assessed how well the left ventricle was functioning ( Fig. 2 ).

**Fig. 3:**
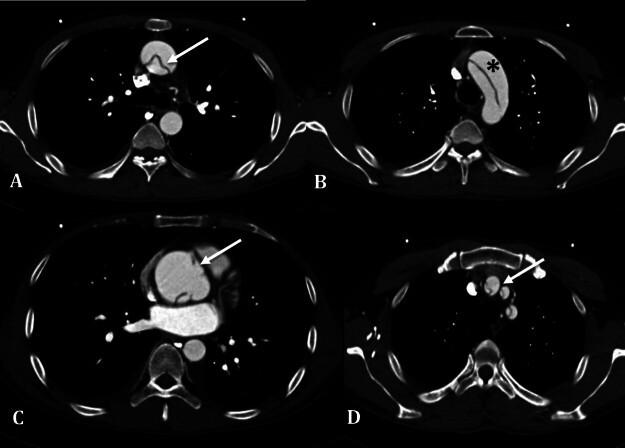
ECG guided CT- Aortogram. ( **A** ) Showing dissection flap at the level of ascending aorta. ( **B** ) Showing dissection flap at the level of arch of aorta. ( **C** ) Showing dissection flap at the level of root of aorta. ( **D** ) Showing dissection flap extending into neck vessels.

**Fig. 4:**
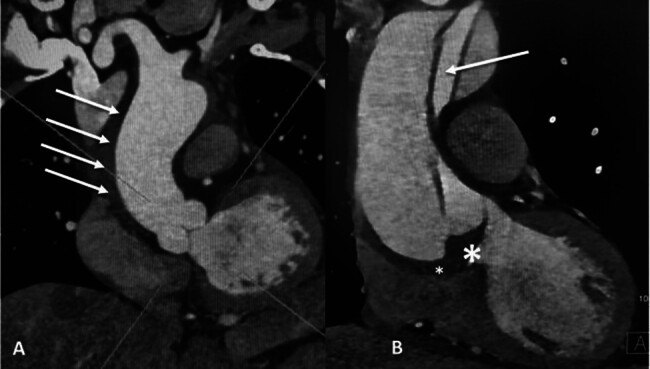
CT Aortogram. ( **A** ) Arrows depicting intramural hematoma. ( **B** ) Astrex depicting intramural hematoma.

The ECG-guided contrast-enhanced computed tomography aortogram with coronary evaluation ( Fig. 3 ), which is part of the emergency protocol, was checked for the sizes of the aorta at specific locations (aortic annulus, aortic sinus, sinotubular junction, proximal ascending aorta, aortic arch opposite to innominate artery, and left subclavian artery, descending thoracic aorta at the level of diaphragm), 12 valvular morphology, coronary artery involvement, the extent of dissection, dissection flap, arch vessel involvement, abdominal vessel involvement, false lumen, intramural hematoma ( Fig. 4A ), pericardial effusion, and pleural effusion.

CT imaging protocol: scan protocol and image reconstruction. We performed all examinations on a SOMATOM FORCE (Siemens Healthcare, Forchheim, Germany) CT scanner. The scanner is a third-generation dual-source CT scanner with two X-ray tubes and corresponding detectors with an angular offset of 90 degrees. They rotate simultaneously, capturing image data in half the time as compared with single-source scanners. The scanner has a temporal resolution as low as 66 ms. Along with increasing the acquisition speed, advanced dose reduction techniques also help reduce the radiation dose by more than half. The tests were done using a method that syncs the start of the imaging process with 60% of the time between heartbeats, as shown on the simulated ECG.

Images were put together using a high-tech method called model-based iterative reconstruction (strength level 3; Siemens Healthcare, Forchheim, Germany) with a medium soft-tissue filter (Bv40) and a slice thickness of 0.6 mm, increasing by 0.4 mm (field of view, 200 mm; pixel matrix, 512 × 512). Axial sections were examined along with coronal and sagittal views and then three-dimensional images and maximum intensity projection images were created on a separate computer system.

**Fig. 5:**
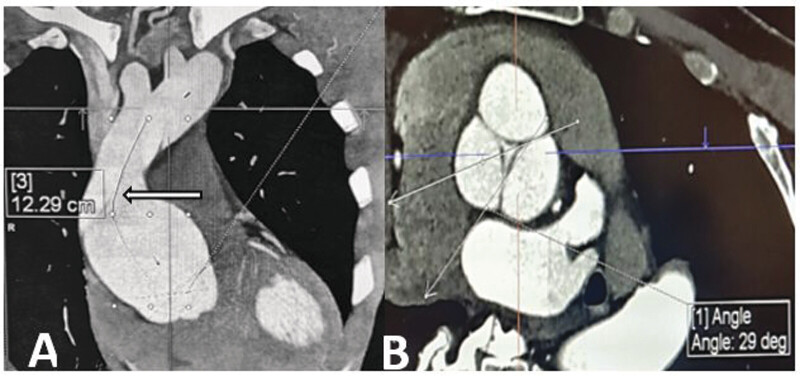
ECG gated CT aortogram. ( **A** ) Used to calculate the length of ascending aorta. ( **B** ) Used to calculate aortic root rotation angle.

We manually marked the aortic annulus and the origin of the innominate artery at the appropriate level and plane when measuring ascending aortic length (AAL). The imaging system could then trace the aorta along the center line automatically and perform curved multiplanar reformatting. We confirmed the anatomical landmarks again on the reconstructed flattened aorta. We then measured the AAL, which is the direct distance along the center line between the annulus and the origin of the innominate artery ( Fig. 5A ). A true short-axis view of the aortic root allowed for the measurement of the aortic root rotation angle. A line between the midpoint of the noncoronary sinus and the anterior commissure was drawn and defined as the “axis of the noncoronary sinus.” Additionally, the “axis of interatrial septum” was created, and the angle formed (either turning to the right or left) was measured and called the aortic root rotation angle 13 ( Fig. 5B ).

### Statistical Analysis

We used univariate analysis to provide frequency and percentage distributions of qualitative demographic and comorbidity variables. Mean ± standard deviation was reported, while for non-normally distributed quantitative variables, median ± (min-max) was reported. Data analysis was performed using the Statistical Package for Social Sciences, version 18.0 (SPSS, Chicago, Ill.).

## Results

### Demographics

A total of 162 patient records were reviewed; 131 (80.8%) patients were male, and 31 (19.2%) were female. The mean age of all patients was 43.3 years (± 13.49). A total of 146 patients (90.1%) had TAAD, and 16 patients (9.8%) had chronic TAAD. 44.4% of all patients had a history of HTN. A history of DM was present in only 3.7% of the patients. An ascending aortic aneurysm was present in 10 patients (6.1%) who presented with TAAD. 27.8% ( *n* = 45) of patients were smokers or had a history of smoking, whereas 17.2% ( *n* = 28) of patients had a history of alcohol intake. Marfan syndrome was present in 27.8% ( *n* = 45) of all patients. Five patients (3.1%) had a history of cerebrovascular disease. ( Table 1 )

**Table 1:** Demographic profile of patients ( *N* = 162)

Features	Number (%)
Age in years (mean ± SD)	43.33 (13.4)
Gender Male Female	131 (80.8) 31 (19.2)
Type of Type A aortic dissection Acute Chronic	146 (90.1) 16 (9.9)
Aortic aneurysm	10 (6.2)
Diabetes mellitus	6 (3.7)
Hypertension	72 (44.4)
Marfan syndrome	45 (27.8)
Smoking tobacco	45 (27.8)
Alcohol intake	28 (17.3)
Previous cerebrovascular accident	5 (3.1)

Abbreviation: SD, standard deviation.

### Presenting Symptoms and Signs

Severe pain was the most common presenting symptom, and 88.9% of patients reported chest pain. The majority of patients ( *n* = 82, 50.6%) complained of anterior chest pain, which was typical in patients with TAAD; 24.7% ( *n* = 40) had both anterior chest pain and back pain, and 1.2% ( *n* = 2) of patients exclusively had back pain. In contrast, no pain was present in 11.1% ( *n* = 18) of patients. Notably, 82.7% ( *n* = 134) described the pain as having a sudden onset. ( Table 2 ) In our center, 54 patients (33.3%) reported within 24 hours of the onset of pain, 45.6% ( *n* = 74) of patients presented between 2 days and 14 days, and 11.1% ( *n* = 14) of patients reported after 14 days of the onset of pain. The majority of patients ( *n* = 130, or 80.3%) had severe pain, whereas only two patients had mild pain. Other presenting complaints comprised palpitations in 26.5% ( *n* = 43) of patients, dyspnea in 42% ( *n* = 68), and syncope in 13.6% ( *n* = 22) of patients at the time of presentation. Seven patients had a past history of cerebrovascular accident (CVA). Of these, three patients had focal neurological deficits at presentation.

**Table 2:** Presenting symptoms and physical findings ( *N* = 162)

Presenting symptoms and physical examination	Number (%)
Pain on presentation Present Absent	144 (88.8) 18 (11.1)
Sudden onset pain	134 (82.7)
Site of pain	
Anterior chest pain	82 (50.6)
Back pain	2 (1.2)
Abdominal pain	4 (2.4)
Absent	14(8.6)
Anterior chest pain + back pain	40(24.6)
Anterior chest pain + abdominal pain	13(8.0)
Anterior chest pain + back pain + abdominal pain	7(4.3)
Severity of pain Mild Moderate Severe	2 (1.2) 12 (7.4) 130 (80.2)
Duration from start of pain to reporting to AIIMS	
0–1 d	54 (33.3)
2–7 d	44 (27.1)
8–14 d	30 (18.5)
Absent	18 (11.1)
> 14 d	16 (9.8)
Severity of pain Mild Moderate Severe	2 (1.2) 12 (7.4) 130 (80.2)
Palpitation	43 (26.5)
Dyspnea	68 (41.9)
Syncope	22 (13.5)
Cerebrovascular accident	7 (4.3)
Hypertension (SBP > 140)	67 (41.3)
Normotensive (SBP = 100–139)	83 (51.2)
Hypotensive (SBP < 100 )	11 (6.7)
Shock (SBP < 80 mm Hg)	1 (0.6)
Peripheral pulse deficit	26 (16.1)
Malperfusion syndromes Renal Lower limb Ischemia CVA	4 (2.4) 23 (14.2) 5 (3.1)

Abbreviation: CVA, cerebrovascular accident; SBP, systolic blood pressure.

More than half (58.7%) of patients with TAAD had initial systolic blood pressure in the range of 100–139 mm Hg, whereas 41.4% ( *n* = 67) had systolic pressures of more than 140 mm Hg. 6.8% ( *n* = 11) of patients had systolic blood pressure less than 100 mm Hg. Documentation revealed a peripheral pulse deficit in 16.1% ( *n* = 26) of patients with TAAD.

### Malperfusion Syndromes

Four patients (2.7%) had renal malperfusion, and 23 (14%) had lower limb ischemia, with 3 having bilateral lower limb ischemia. Five patients had cerebrovascular accidents of new onset on presentation

### Initial Investigations

Chest radiography showed mediastinal widening in 143 patients (88.8%). 11.2% of patients showed no chest radiography abnormalities.

### Diagnostic Imaging

Out of 143 patients who had transthoracic echocardiography (TTE), 87% had a widened ascending aorta, and 81.4% of them showed signs of dissection flaps. ( Table 3 ). The majority of patients ( *n* = 155, 96.3%) had aortic valve regurgitation, severe in 105 (65.2%) and moderate in 20 (12.4%). Five patients had severe mitral regurgitation. One patient had significant aortic stenosis. Severe left ventricular dysfunction was present in 28 patients (17.4%), and moderate dysfunction in 16 patients (10%). Pericardial effusion was present in 41 (25.5%) patients. The mean aortic root diameter was 30.6 mm (± 7.4 mm), with the minimum being 17 mm and the maximum being 70 mm.

**Table 3:** Investigations: chest X-ray, two-dimensional transthoracic echocardiography

Investigation	*N* *(%)
Chest X-ray—mediastinal widening	143 (88.8)
Echocardiography	
Ascending aorta > 4.5 cm	140 (86.9)
Dissection flap presence	131 (81.3)
Bicuspid aortic valve	12 (7.4)
Severe aortic regurgitation	105 (65.2)
Left ventricle dysfunction Mild Moderate Severe	28 (17.3) 16 (9.9) 22 (13.6)
Pericardial effusion Mild Moderate Severe	30 (18.6) 8 (4.9) 3 (1.8)

### ECG Gated Computed Tomography Angiography Dimensions

Aortic valve annulus mean diameter was 31.5 mm (± 7.1), with the maximum diameter being 50 mm and minimum diameter being 16 mm ( Table 4 ) The mean aorta diameter at the sinus level was 50.6 mm (± 11.9), with the maximum diameter being 84 mm and the minimum diameter being 28 mm. In 109 (67.2%) patients, the sinotubular junction was effaced. The mean ascending aorta diameter beyond the sino-tubular junction was 54.5 mm (± 15.8), with the maximum diameter being 100 mm and the minimum diameter being 26 mm. The mean aortic arch diameter opposite the innominate artery was 29.1mm (± 6.4 mm), with the maximum diameter being 66 mm and the minimum diameter being 15 mm. The mean diameter of the descending thoracic aorta at the diaphragm level was 25.1 mm (±6.9 mm), with the maximum diameter being 67 mm and the minimum diameter being 15 mm. In 143 patients, the mean true lumen diameter was 17.9 mm (± 11.3 mm), with the maximum diameter being 94 mm and the minimum being 7 mm, and the mean false lumen diameter was 39 mm (± 13.7 mm), with a maximum diameter of 85 mm and a minimum diameter of 4 mm. In 146 patients, the aortic root rotation angle was calculated, with a mean of +26.0 degrees (± 10.0 degrees), with the maximum angle being +52 degrees and the minimum angle being +2 degrees. In 143 patients, the aortic length (from aortic annulus until The origin of the innominate artery was measured, with the mean length being 118.1 mm (± 17.7 mm), the maximum aortic length being 182 mm, and the shortest length being 88 mm.

**Table 4:** Computed tomography angiography: findings limited to intrapericardial aorta

ECG-gated CT aortography—intrapericardial aorta	
Dimensions	
Aortic annulus mean (SD) mm	31.5 (7.1)
Sinus segment mean (SD) mm	50.6 (11.9)
Ascending aorta mean (SD) mm	54.5 (15.7)
Aortic arch opposite to innominate artery mean (SD) mm	29.1 (6.4)
DTA mean (SD) mm	25.1 (6.9)
Aortic root angle mean (SD) mm	26.1 (10.1)
Ascending aorta length mean (SD) mm	118.1 (17.7)
Entry tear location Annulus to STJ STJ to brachiocephalic trunk No tear	94 (58.7) 50 (31.2) 16 (10.0)
Bicuspid aortic valve	14 (8.6)
Left main coronary artery True Lumen origin False lumen origin Dissection	158 (98.7) 2 (1.3) 6 (3.7)
Right coronary artery True lumen origin False lumen origin Dissection	130 (81.2) 30 (18.7) 18 (11.2)
Pericardial effusion Mild Moderate Severe	13 (8.1) 10 (6.3) 3 (1.8)
Primary tear morphology Longitudinal Horizontal Oblique Circumferential	78 (54.5) 50 (34.9) 13 (9.1) 2 (1.4)

Abbreviation: CT, computed tomography; DTA, descending thoracic aorta; ECG, electrocardiogram; SD, standard deviation; STJ, sinotubular junction.

Common carotid arteries (CCA) were evaluated in 147 patients. Dissection was found in 46 (31.3%) patients, with 25.85% ( *n* = 38) being in the proximal common carotid artery, 4.76% ( *n* = 7) being in the middle common carotid artery, and 0.6% ( *n* = 1) being in the distal part. The left-sided common carotid artery was involved in 20 (13.6%) patients, the right side in 18 (12.2%), and bilateral involvement was present in 8 patients (5.4%). Six patients had an intramural hematoma, and 4 patients had a penetrating aortic ulcer.

Primary tear morphology was found to be longitudinal in 78 (54.5%) patients, horizontal in 50 (35%), oblique in 13 (9.0%) patients, and circumferential in 2 patients. Out of 162 patients, 8.6% ( *n* = 14) had a bicuspid aortic valve, whereas the rest, 91.4% ( *n* = 148), had a tricuspid valve. The left main coronary artery was seen arising from the false lumen in 2 cases, whereas 6 patients had dissection extending into a left main coronary artery. The left anterior descending artery was diseased in five patients, with no disease in the left circumflex artery. The right coronary artery arose from the false lumen in 18.8% ( *n* = 30) of patients, with dissection extending into the right coronary artery in 11.3% ( *n* = 18) of patients. Pericardial effusion was found in 26 (16.3%) patients. Entry tear location was analyzed, and it was found that 16 (10%) had no visible entry tear. In 94 (58.8%) patients, there was an entry tear located between the aortic valve and the sinotubular junction, while 50 (31.3%) patients had an entry tear between the sinotubular junction and the innominate artery. Dissection was only in the ascending aorta for 10 (6.3%) patients, extended to the arch for 25 (13.8%) patients, went to the descending thoracic aorta for 45 (28.1%), reached the abdominal aorta for 11 (6.9%), and got to the aortic bifurcation for 22 (13.8%) patients. In 46 (28.8%), dissection reached even the common iliac arteries. Arch vessel dissection was present in 90 (61.2%) patients ( Table 5 ). The false lumen was patent in 132 (82.5%) patients, thrombosed in 15 (9.4%), and partially thrombosed in 13 (8.1%) patients.

**Table 5:** Computed tomography angiography: findings limited to extrapericardial aorta and its branches

ECG-gated CT aortography—extrapericardial aorta and its branches	
Distal extent of dissection Ascending aorta Arch DTA Abdominal aorta Bifurcation of abdominal aorta Common iliac artery	10 (6.2) 25 (15.6) 45 (28.1) 11 (6.9) 22 (13.7) 46 (0.6)
Arch vessel involvement Innominate artery Left CCA Left SCA Innominate + left SCA All arch vessels No involvement	32 (21.7) 20 (13.6) 9 (6.1) 2 (1.3) 27 (18.3) 57 (38.7)
Common carotid artery dissection Proximal Middle Distal Left CCA Right CCA B/L involvement	38 (25.8) 7 (4.7) 1 (0.6) 20 (13.6) 18 (12.2) 8 (5.4)
Penetrating aortic ulceration	4 (2.7)
Intramural hematoma	6 (4.1)
Celiac artery True lumen False lumen	130 (80.7) 31 (19.2)
Superior mesenteric artery True lumen False lumen	145 (90.6) 15 (9.4)
Inferior mesenteric artery True lumen False lumen	152 (95.6) 7 (4.4)
Left renal artery True lumen False lumen	129 (80.6) 31 (19.4)
Right renal artery True lumen False lumen	138 (86.8) 21 (13.2)
False lumen Patent Thrombosed Partially thrombosed	132 (82.5) 15 (9.4) 13 (8.13)

Abbreviations: B/L, bilateral; CCA, common carotid artery; CT, computed tomography; DTA, descending thoracic aorta; ECG, electrocardiogram; SCA, superior cerebellar artery.

In 21 (13.2%) patients, the right renal artery was arising from the false lumen, whereas the left renal artery arose from the false lumen in 31 (19.4%) patients. Only 4 (2.7%) patients had features of renal malperfusion. The celiac artery arose from the false lumen in 31 (19.3%) patients, and the superior mesenteric artery arose from the false lumen in 9.4% ( *n* = 15) of patients. However, none of the patients had documented clinically significant mesenteric ischemia. CT angiography examination revealed pleural effusion in 56 out of 160 patients (35%).

## Discussion

The high mortality and lack of antemortem diagnosis are major problems with TAAD. Delay in the accurate diagnosis is a major contributing factor to the consistently high mortality of aortic dissection, despite significant progress in diagnostic and treatment skills. 14 Most of the untreated patients with proximal aortic dissection and half of those with distal dissection die within one year, usually within the first two weeks. Death is usually caused by acute aortic regurgitation, branch vessel obstruction, or aortic rupture; the risk of a fatal rupture from untreated ascending aorta dissection is around 90%. Long-term survival of aortic dissection in patients who undergo surgical repair and survive long enough to leave the hospital is currently 90% and 88% at 5 and 10 years, respectively. 15

In our study, we discovered that TAAD happened more often in men, with 4.2 men for every woman, while the International Registry for Aortic Dissection (IRAD) 4 16 showed a ratio of 1.88 to 1, Hungary had 1.7 to 1, and Taiwan had 2.17 to 1; however, it was close to studies from China, which reported a ratio of 3.55 to 1. 17 The difference might have been because the men in India had more risk factors. For example, smoking is an important risk factor. In India, the smoking rate in men was much greater than that in women (14% vs 1%), 18 and in IRAD countries, the rates were similar between men and women (30% vs 20%). This difference might result in a predominance of TAAD in male Indian patients. Additionally, other risk factors, such as HTN and atherosclerosis, are associated with a high prevalence of TAAD in male patients. In the Japan Registry of Aortic Dissection (JRAD), the mean age of all patients was 67.9 years, and 584 (47.9%) of the patients were male. A total of 241 patients (19.8%) were 80 years old or older. 19 In our study, the mean age was 43.3 years (± 13.49 years), which was lower than the described mean age in the IRAD of 63.1 years 16 but aligned with Yang Li et al.'s study in Chinese patients with a mean age of 47.5 years ± 11.2 years. Accordingly, we inferred that early onset might be a feature of TAAD in India; however, the cause of the onset is still unclear. This variation may be due to a larger proportion of patients in our study with connective tissue disorders presenting early. Furthermore, other factors, such as race and the lack of routine clinic visits, were taken into account, although no systematic epidemiologic survey was performed.

The crux of a successful outcome in aortic dissection is an early diagnosis and prompt administration of the appropriate treatment. The most common initial clinical presentation of aortic dissection is pain, but as many as 5–10% of dissections remain painless and, therefore, cause diagnostic dilemmas and delays in treatment 20 According to IRADs, 85.4% of patients had an abrupt onset of pain, and 12.7% had syncope. 16 In our study, pain was also the most common presenting symptom, with 89% of patients reporting it, but 11.1% had no pain. Similar to the reported literature, syncope was present in 13.6% of patients.

The median referral interval from the onset of symptoms to arrival at JRAD centers was 3.4 hours (range: 0 to 292 hours). 19 Sixty-seven percent of all patients either directly went to JRAD centers or received transportation from hospitals within 6 hours of the onset of symptoms. Almost all patients transferred to JRAD hospitals (81.1%) arrived within 2 hours. In our study, almost one-third ( *n* = 54) of patients reported to our center within 24 hours of the onset of pain; however, 45.6% ( *n* = 74) presented between 2 and 14 days, and 11.1% ( *n* = 14) reported after more than 14 days. This delay in presentation can be attributed to a lack of awareness about the condition in general populations as well as doctors leading to late referrals, a lack of adequate healthcare infrastructure to cater to the condition leading to increased travel time to super- specialty centers, and an uneducated and poor population.

Aortic dissection has a diversity of presentations. Therefore, doctors should be especially alert for aortic dissection in patients who have risk factors, such as high blood pressure, aortic aneurysms, or family history of connective tissue disorders. Usually, the patient is a man in his 60s with high blood pressure who suddenly feels chest pain and has risk factors like connective tissue disorders (such as Marfan's syndrome, Turner syndrome, or Ehlers-Danlos syndrome), an aortic aneurysm, or other heart-related issues. 21

Hagan PG et al., in their study of 289 patients with TAAD, found that a history of HTN was elicited in 69.3% of all patients with TAAD. DM was present in 3.9% of patients, Marfan syndrome was present in 6.7% of patients, and 12.4% were known cases of aortic aneurysm. 16

In a more extensive study published by IRADs with 2,952 patients with TAAD, a history of HTN was elicited in 74.4% of all patients of TAAD, DM was present in 7.7% of patients, Marfan syndrome was present in 4% of patients, 12.7% were known case of aortic aneurysm 22 In JRAD, 63.1% of patients had a history of HTN, and 2.2% had Marfan syndrome. 19 In our study, we found that 44.4% of all patients had a history of HTN. This increase was probably due to the younger patient population. A history of the presence of DM was present in only 3.70% of patients. In 6.2% of patients, a history of ascending aortic aneurysm was present in those presenting with TAAD. Forty-five patients (27.8%) were smokers or had a history of smoking, whereas 17.28% ( *n* = 28) had a history of alcohol intake. In contrast to the Western literature, Marfan syndrome was present in 27.8% ( *n* = 45) of all patients.

Chest radiographic findings may be normal in 10% to 40% of aortic dissections. A study noted a widened mediastinum in 61.1% of cases of aortic dissection. 14.1% of cases reported displacement of aortic calcification, and 25.8% noted an abnormal cardiac contour. 16 In JRAD Chest roentgenograms were used to evaluate 920 patients (75.6%), and the most common finding was a widened mediastinum in 561 (61.0%) 19 In our study, chest radiography showed the presence of mediastinal widening in 88.82% (143) of patients with type A dissection. We observed no chest radiography abnormalities in 11.2% of the patients.Transthoracic echocardiography (TTE) is a non-invasive examination that may help detect an ascending aortic dissection flap. It has a reported sensitivity of 59–83% and a specificity of 63–93% for the diagnosis of aortic dissection. The sensitivity of transthoracic echocardiography is between 78% and 100% for the diagnosis of a type A dissection. 23 24 25 TTE is not very helpful for looking at the whole aorta, but it is excellent for spotting problems with the aortic valve, pericardial tamponade, or issues with heart wall movement, and it can check for aortic dissection in patients with shock in the emergency room. However, it has trouble seeing the far parts of the ascending and transverse aorta. It is limited, however, in visualizing the distal ascending and transverse aorta. 26 In our study, similar to the reported literature, transthoracic echocardiography could diagnose a dissection flap in 81.4% of cases.

When suspecting aortic dissection, CT is typically the preferred imaging modality. computed tomography angiography (CTA) is very accurate, with close to 100% sensitivity and specificity 27 ; has the advantage of being easily accessible in most medical settings 6 ; can be programmed to provide a rapid acquisition of images, allowing for a quick diagnosis 28 ; and usually is able to demonstrate well the extent of the dissection. At CTA, ECG gating helps clearly show how far the mural flap extends near the aortic valve and coronary arteries, which is important because it prevents doctors from misdiagnosing aortic dissections by confusing a motion blur with a mural flap. In practice, cardiac gating has become a standard procedure for CT angiography in suspected aortic dissections. 29

Current guidelines recommend surgery when the ascending aorta size reaches 5.5 cm for non-syndromic patients and 4.5 cm in syndromic patients. 30 However, data from the International Registry of Acute Aortic Dissections showed that aortas could dissect at smaller sizes than that advocated in the guidelines. Among the 591 cases of type A TAD, 59% occurred at sizes less than 5.5 cm, and 40% occurred at sizes less than 5.0 cm. 31 These data correspond with our center's experience. An ascending aorta diameter of less than 55 mm was seen in 59.9% (97) of patients, with only 40.12% (65) of patients having an ascending aorta diameter equal to or more than 55 mm.

In 2,137 consecutive patients enrolled in GERAADA (the German Registry for Acute Aortic Dissection Type A), 37.3% had supra-aortic branch extensions of TAAD. 32

The Penn-Saitama-Freiburg registry for CCA dissection in TAAD included 1,444 patients without Marfan syndrome who had CT scans before surgery and were treated for TAAD between 2002 and 2017. In this study, 1,004 patients (70%) did not have any CCA dissection, 279 patients (19%) had CCA dissection on one side, and 161 patients (11%) had CCA dissection on both sides. In this study, a total of 1,004 patients (70%) did not have any CCA dissection, 279 patients (19%) presented with unilateral CCA dissection, and 161 patients (11%) had bilateral CCA dissection. Since there were no major differences between patients with one-sided or both-sided CCA dissection, the study looked at the clinical features, imaging results, and hospital and long-term outcomes of patients without CCA dissection compared with those with CCA dissection (440 patients). 33 In our study, we found that in 61.22% (90) of patients, the dissection affected the arch vessels. The group of patients included 21.76% (32) who had issues with the innominate artery, 13.60% (20) with the left common carotid artery, 6.12% (9) with the left subclavian artery, 18.36% (27) with all the arch vessels, and 1.36% (2) with both the innominate and left subclavian arteries. Knowledge of CCA dissection is important for planning a cannulation strategy at the time of surgical intervention.

A recent study has shown a relationship between the increased clockwise rotation of the aortic root and dilation of the ascending aorta. 4 16 17 Researchers have raised the presumption that unusual aortic root rotation angles could potentially contribute to dissection. Researchers independently found a direct association between the aortic root rotation angle and thoracic aortic dissection. 13 Moradi et al., in their study the mean of rotation angle in the case group of 25 patients with type A aortic, The average aortic root rotation angle and aortic diameter in the 25 patients were 22.5 ± 10.5° and 43.1 ± 12.5 mm, while in the control group, they were 15.7 ± 10.7° and 30.7 ± 5.3 mm (P-value = 0.007 and 0.001, respectively). A The study found a direct relation between the aortic root rotation angle and aortic diameter (P-value = 0.007, r = 0.276). Their study concluded that there was an independent association between the aortic root rotation angle and thoracic aortic dissection. 34 In our study, the aortic root rotation angle was calculated in 146 patients, with a mean of +26.0 degrees (± 10.0 degrees), the maximum angle being +52 degrees, and the minimum angle being +2 degrees. The higher aortic root angle in our study can be attributed to the higher number of patients having dilated aortic roots due to connective tissue disorders.

Jinlinwu et al., 12 in their study of 522 patients, found the average aortic diameter to be 4.8 cm ± 0.7 (range: 3.5–9 cm) and the ascending aorta length to be 11.2 ± 1.3 cm (range 7.3 to 15.4 cm). Of the 522 patients, 64 experienced aortic dissection. Research indicates that when dissection occurs, the aortic diameter increases by 16.9% to 31.9%. 35 Therefore, previous studies may have overestimated the diameter cutoff, suggesting a “leftward shift” in the intervention standard. Surprisingly, the increase in ascending aorta length after dissection was only 2.7%, which was similar to another study (5.4% by Rylski et al.). Regarding the prior aortic size, time interval, age, and sex, the sudden increase caused by dissection per se is even closer to zero (p > 0.05). The consistent size of the ascending aorta during aortic dissection helps in determining the right point for treatment without being affected by any sudden growth that might happen right after the dissection. In our study, in 143 patients with TAAD, the aortic length (from aortic annulus until origin of the innominate artery) was measured, with the mean length being 11.8 cm (± 1.7 cm), with the maximum aortic length being 18.2 cm and the shortest length being 8.8 cm. Since in our study the majority of patients had an ascending aorta diameter less than 5.5 cm, the cutoff limit for surgical intervention, there is a need for other dimensions that can be considered as markers for early surgical intervention to avoid mortality and morbidity. In their study, Tobias Kruger et al. 35 stated that increasing AAL is a potential risk factor for aortic dissection. We can use AAL in conjunction with ascending aorta diameter to subject patients to early interventions. We cannot comment much on the significance of ascending aorta length in the Indian population, as there is a lack of data on AAL in the undissected Indian population for comparison.

### Study Limitation

Although our data provide an outline of TAAD in India, our study's retrospective observational design is a major limitation. TA Another limitation of this study was its small sample size. Therefore, we need multicenter, prospective, large-scale studies to fully elucidate the clinical situation of TAAD in India. This study does not accurately reflect the current situation, as only survivors were able to access our center. There is no information available about the patients who succumbed undiagnosed or those who could not reach the hospital.

## Conclusion

We analyzed the clinical characteristics and radiological features of TAAD in our center over the past 10 years. We also compared our data with those reported by IRAD, JRAD, and GERAADA. Acute chest pain was the most common presenting complaint. We found that, compared with Western populations, Indian patients with TAAD had delayed reporting to health care facilities, an early onset, more male patients, a low incidence of HTN, and a higher incidence of Marfan patients. CT angiography was the investigation of choice, with TTE being a helpful support tool. The majority of our patients had TAAD, despite their aortic diameter being less than 55 mm. Our demographic data and clinical presentation were similar to Chinese data, owing to similar socioeconomic backgrounds.

In India, it is necessary to conduct a large-scale study to determine the gravity of the situation and alleviate the existing void in the diagnosis and management of TAAD.

## References

[1] Criado F J Aortic dissection: a 250-year perspective Tex Heart Inst J 2011 38 06 694 700 22199439 PMC3233335

[2] Levy D Goyal A Grigorova Y Aortic dissection StatPearls Internet 2020

[3] Spittell P C Spittell J A Jr Joyce J W Clinical features and differential diagnosis of aortic dissection: experience with 236 cases (1980 through 1990) Mayo Clin Proc 1993 68 07 642 651 10.1016/s0025-6196(12)60599-0 8350637

[4] Mészáros I Mórocz J Szlávi J Epidemiology and clinicopathology of aortic dissection Chest 2000 117 05 1271 1278 10.1378/chest.117.5.1271 10807810

[5] Hirst A E Jr Johns V J Jr Kime S W Jr Dissecting aneurysm of the aorta: a review of 505 cases Medicine (Baltimore) 1958 37 03 217 279 13577293 10.1097/00005792-195809000-00003

[6] McMahon M A Squirrell C A Multidetector CT of aortic dissection: a pictorial review Radiographics 2010 30 02 445 460 10.1148/rg.302095104 20228328

[7] Erbel R Alfonso F Boileau C Task Force on Aortic Dissection, European Society of Cardiology Diagn Manag Aortic Dissection Eur Heart J. 2001 22 1642 1681 10.1053/euhj.2001.2782 11511117

[8] von Kodolitsch Y Schwartz A G Nienaber C A Clinical prediction of acute aortic dissection Arch Intern Med 2000 160 19 2977 2982 10.1001/archinte.160.19.2977Abstract 11041906

[9] Lai V Tsang W K Chan W C Yeung T W Diagnostic accuracy of mediastinal width measurement on posteroanterior and anteroposterior chest radiographs in the depiction of acute nontraumatic thoracic aortic dissection Emerg Radiol 2012 19 04 309 315 10.1007/s10140-012-1034-3 22415593 PMC3396328

[10] Funakoshi H Mizobe M Homma Y Nakashima Y Takahashi J Shiga T The diagnostic accuracy of the mediastinal width on supine anteroposterior chest radiographs with nontraumatic Stanford type A acute aortic dissection J Gen Fam Med 2018 19 02 45 49 10.1002/jgf2.155 29600127 PMC5867066

[11] Masri A Kalahasti V Svensson L G Aortic cross-sectional area/height ratio and outcomes in patients with a trileaflet aortic valve and a dilated aorta Circulation 2016 134 22 1724 1737 27770001 10.1161/CIRCULATIONAHA.116.022995

[12] Wu J Zafar M A Li Y Ascending aortic length and risk of aortic adverse events: the neglected dimension J Am Coll Cardiol 2019 74 15 1883 1894 10.1161/CIRCULATIONAHA.116.022995 31526537

[13] Moradi M Mirfasihi R S Is there any association between aortic root rotation angle and aortic dissection? Indian J Thorac Cardiovasc Surg 2019 1 5 10.1007/s12055-019-00859-2 PMC7525551 33061123

[14] Crawford E S The diagnosis and management of aortic dissection JAMA 1990 264 19 2537 2541 10.1001/jama.1990.03450190069031 2232021

[15] International Registry of Acute Aortic Dissection (IRAD) Tsai T T Evangelista A Nienaber C A Long-term survival in patients presenting with type A acute aortic dissection: insights from the International Registry of Acute Aortic Dissection (IRAD) Circulation 2006 114(1, Suppl)I350 I356 10.1161/CIRCULATIONAHA.105.000497 16820599

[16] Hagan P G Nienaber C A Isselbacher E M The International Registry of Acute Aortic Dissection (IRAD): new insights into an old disease JAMA 2000 283 07 897 903 10.1001/jama.283.7.897 10685714

[17] Yu H-Y Chen Y-S Huang S-C Wang S S Lin F Y Late outcome of patients with aortic dissection: study of a national database Eur J Cardiothorac Surg 2004 25 05 683 690 10.1016/j.ejcts.2003.12.041 15082267

[18] India - tobacco smoking rate by gender and type 2017 | Statista [Internet]. [cited. (202124).https://www.statista.com/statistics/865496/india-tobacco-smoking-rate-by-gender-and-type/

[19] Inoue Y Matsuda H Uchida K Analysis of acute type A aortic dissection in Japan registry of aortic dissection (JRAD) Ann Thorac Surg 2020 110 03 790 798 32035913 10.1016/j.athoracsur.2019.12.051

[20] Liu Z Y Zou Y L Chai B L Zeng H S Analysis of clinical features of painless aortic dissection J Huazhong Univ Sci Technolog Med Sci 2014 34 04 582 585 25135731 10.1007/s11596-014-1319-8

[21] Hebballi R Swanevelder J Diagnosis and management of aortic dissection Contin Educ Anaesth Crit Care Pain 2009 9 14 18

[22] Pape L A Awais M Woznicki E M Presentation, diagnosis, and outcomes of acute aortic dissection: 17-year trends from the International Registry of Acute Aortic Dissection J Am Coll Cardiol 2015 66 04 350 358 26205591 10.1016/j.jacc.2015.05.029

[23] Daily P O Trueblood H W Stinson E B Wuerflein R D Shumway N E Management of acute aortic dissections Ann Thorac Surg 1970 10 03 237 247 5458238 10.1016/s0003-4975(10)65594-4

[24] European Association of Echocardiography Document Reviewers Evangelista A Flachskampf F A Erbel R Echocardiography in aortic diseases: EAE recommendations for clinical practice Eur J Echocardiogr 2010 11 08 645 658 20823280 10.1093/ejechocard/jeq056

[25] Meredith E L Masani N D Echocardiography in the emergency assessment of acute aortic syndromes Eur J Echocardiogr 2009 10 01 i31 i39 19131497 10.1093/ejechocard/jen251

[26] Kim Y W Park Y J Kim D K Optimal Imaging for Aortic DissectionEndovasc Today. November 2015, 5

[27] Sentz A The role of CTA, MRA, and sonography in aortic dissection J Diagn Med Sonogr 2015 31 235 240

[28] Braverman A C Acute aortic dissection: clinician update Circulation 2010 122 02 184 188 20625143 10.1161/CIRCULATIONAHA.110.958975

[29] Grabenwoger M Weiss G Type A aortic dissection: the extent of surgical intervention Ann Cardiothorac Surg 2013 2 02 212 215 23977585 10.3978/j.issn.2225-319X.2013.02.03 PMC3741842

[30] Mokashi S A Svensson L G Guidelines for the management of thoracic aortic disease in 2017 Gen Thorac Cardiovasc Surg 2019 67 01 59 65 29030719 10.1007/s11748-017-0831-8

[31] International Registry of Acute Aortic Dissection (IRAD) Investigators Pape L A Tsai T T Isselbacher E M Aortic diameter >or = 5.5 cm is not a good predictor of type A aortic dissection: observations from the International Registry of Acute Aortic Dissection (IRAD) Circulation 2007 116 10 1120 1127 17709637 10.1161/CIRCULATIONAHA.107.702720

[32] Czerny M Schoenhoff F Etz C The impact of pre-operative malperfusion on outcome in acute type A aortic dissection: results from the GERAADA registry J Am Coll Cardiol 2015 65 24 2628 2635 26088302 10.1016/j.jacc.2015.04.030

[33] Kreibich M Rylski B Czerny M Impact of carotid artery involvement in type A aortic dissection Circulation 2019 139 16 1977 1978 30986110 10.1161/CIRCULATIONAHA.118.038099

[34] Saremi F Cen S Tayari N A correlative study of aortic valve rotation angle and thoracic aortic sizes using ECG gated CT angiography Eur J Radiol 2017 89 60 66 28267550 10.1016/j.ejrad.2017.01.009

[35] Krüger T Forkavets O Veseli K Ascending aortic elongation and the risk of dissection Eur J Cardiothorac Surg 2016 50 02 241 247 26984982 10.1093/ejcts/ezw025

